# Food-Induced Emotional Resonance Improves Emotion Recognition

**DOI:** 10.1371/journal.pone.0167462

**Published:** 2016-12-14

**Authors:** Elisa Pandolfi, Riccardo Sacripante, Flavia Cardini

**Affiliations:** Department of Psychology, Anglia Ruskin University, Cambridge, United Kingdom; Universita degli Studi di Udine, ITALY

## Abstract

The effect of food substances on emotional states has been widely investigated, showing, for example, that eating chocolate is able to reduce negative mood. Here, for the first time, we have shown that the consumption of specific food substances is not only able to induce particular emotional states, but more importantly, to facilitate recognition of corresponding emotional facial expressions in others. Participants were asked to perform an emotion recognition task before and after eating either a piece of chocolate or a small amount of fish sauce—which we expected to induce happiness or disgust, respectively. Our results showed that being in a specific emotional state improves recognition of the corresponding emotional facial expression. Indeed, eating chocolate improved recognition of happy faces, while disgusted expressions were more readily recognized after eating fish sauce. In line with the embodied account of emotion understanding, we suggest that people are better at inferring the emotional state of others when their own emotional state resonates with the observed one.

## Introduction

The ability to effectively communicate our emotional states is critical in face-to-face interactions. Emotional states can be either expressed directly through speech or signalled indirectly through non-verbal cues [1,2]. Amongst the possible non-verbal cues, emotional facial expressions are considered the most immediate and effective way to show our emotional states [3].

According to the recent embodied account of emotion understanding [4–6], in order to effectively infer others’ feelings from their facial expressions, we implicitly “simulate” the observed bodily state by generating representations of how we would feel when displaying that particular emotion within our own sensorimotor cortices. Additionally, facial muscles responsible for expression-specific motor repertoires—for example, the zygomatic major activated when smiling or the corrugator supercilii when frowning [7]–are spontaneously activated by mere vision of emotional expressions [8–10].

Results from neuropsychological, behavioural and neuroimaging studies provide support for the embodied account of emotion understanding. Two patients with lesions affecting the insula and neighbouring areas—which are recruited when we are exposed to disgusting odours or tastes—showed an impaired ability to experience disgust, as well as impaired recognition of disgusted facial expressions [1,11]. Interestingly, behavioural results from healthy participants demonstrated that interfering with the facial muscles normally recruited when displaying specific emotions affected recognition of the corresponding facial expressions [12]. Finally, fMRI data showed that brain regions activated during the experience of an emotion are also recruited when observing the corresponding facial expression in others [13].

Importantly, it has been suggested that the relationship between facial mimicry and the processing of others’ emotional facial expressions is reciprocal. In particular, the automatic simulation of an observed facial expression triggers afferent inputs from the receptors activated during facial movements. This eventually induces an emotional state in the observer that can influence his perception of the observed emotional expression [14,16].

Several studies have so far demonstrated that one’s own emotional expression can shape the processing of emotional stimuli and, more importantly, of emotional expressions observed in others, modulating one’s own attitude towards them [14–17]. For example, Laird asked participants to contract specific facial muscles—either the zygomatic major or the corrugator supercilii—therefore producing either a smile or frown. Then participants were asked to provide subjective reports of their mood and to rate the funniness of some cartoons. Participants who smiled rated their mood as happier and the cartoons as funnier than those who were asked to contract their eyebrows, producing a frown [15]. In a more recent study, Khun and colleagues [17] asked participants to recall either a happy or a sad event in their life and then to adopt the corresponding facial expression. Further, participants were presented with different faces, depicting either a happy or a sad expression, and then were asked to report how close they felt to that person. Results showed that faces depicting an emotion compatible with the participant’s mood and facial expression were rated as closer than faces showing an incompatible emotion.

Interestingly, Sel and colleagues tested the effect of merely adopting a specific facial expression on early visual evoked potentials during observation of other people’s facial expressions [18], providing the first electrophysiological confirmation of the top-down modulatory effect of one’s own facial expression on the visual processing of observed emotions. In particular, the authors found that adopting a happy facial expression influenced the perception of neutral faces, so that they were processed similarly to happy faces. This effect was observed in the N170/VPP component, indicating that it occurred at a very early stage of the visual processing of facial emotional expressions.

These previous studies, however, did not explicitly measure whether the experimental manipulation was effective in changing participants’ subjective mood states. A recent study overcame this limitation, by inducing different moods, gathering participants’ subjective reports of their emotional states and, finally, assessing their facial expression recognition abilities [19]. With their study, Schmid and Mast aimed to expand the mood-congruity theory [20,21] to emotion processing, by partially confirming the mood-congruity effect. According to this theory, being in a negative mood facilitates recall of negative events and promotes more negative judgments of other people; conversely, positive moods improve recall of pleasant stimuli and promote positive judgments of others. After inducing either a sad or a happy mood in their participants, Schmid and Mast found that people in a sad mood recognized sad faces better than happy faces. However, this effect was not observed for people in a happy mood. More importantly, further analyses demonstrated that being in an emotional state congruent with the observed one did not improve recognition of that emotion; rather, their results demonstrated that being in a particular mood *hinders* recognition of an incongruent emotional expression [19].

Taken together, these results highlight the need to test whether an effective simulation mechanism requires the observer *to share* the inferred emotion with the other person in order to improve interpersonal emotional understanding. With the present study, we directly tested this suggestion by investigating whether recognition of specific emotions improves when the observer’s emotional state resonates with them.

Food can robustly affect emotional states [22,23]. For example, participants reported increases in positive mood after eating chocolate or an apple [24]. Eating chocolate also was found to improve participants’ experimentally induced negative mood [25]. Finally, participants associated happiness and surprise with a sweet-tasting solution more often than with salty, sour or bitter solutions, whereas the bitter solution was associated with disgust [26].

In line with previous evidence, we provided participants with a small amount of chocolate or fish sauce to induce happiness or disgust, respectively, and then we measured any changes in their emotion recognition abilities. If inducing self-other emotional resonance through a *sharing* mechanism really does play a critical role in emotion understanding, then participants should be better at detecting the emotional state they are in. Therefore, the present study tested—and confirmed—the hypothesis that participants who ate chocolate would be better at recognizing happy faces, while those who ate fish sauce would show an improved ability to recognize disgusted expressions.

## Materials and Methods

### Participants

**Fig 1:**
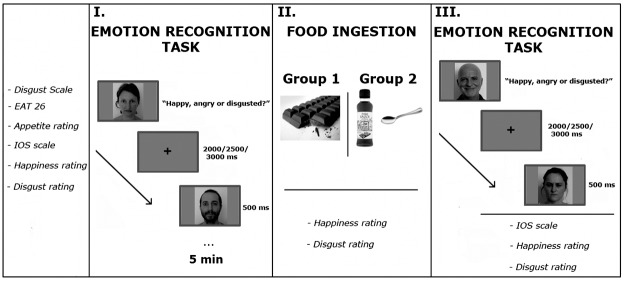
Visual representation of the Experimental Procedure. In an initial phase participants were asked to fill out two questionnaires and to perform four rating judgments. **I.** First session of the Emotion Recognition Task. **II.** Food ingestion: one group of participants was asked to eat ~5gr of dark chocolate, whereas the other group was asked to ingest a spoonful of fish sauce. After food intake participants were asked to perform an emotion intensity judgment. **III.** Second session of the Emotion Recognition Task, followed by another emotion intensity judgment and IOS rating scale. The faces depicted in this figure are not the original ones from the KDEF dataset, but are mere examples. The individuals have given written informed consent (as outlined in PLOS consent form) to publish their faces.

The present research involved human participants and has been approved by the local ethical committee—i.e. the Faculty Research Ethics Panel, at Anglia Ruskin University—and has been conducted according to the principles expressed in the Declaration of Helsinki. Written informed consent was obtained from the participants. Also the four participants depicted in Fig 1 have given written informed consent (as outlined in PLOS consent form) to publish their faces. Fifty healthy volunteers (29 female) between the age of 18 and 53 (M_age_ = 27.02, SD = 8.23) participated in this study—in exchange for £8 Amazon voucher. Participants had normal or corrected to normal vision and reported no food allergies. The sample size was *a priori* decided based on similar psychophysical experiments on emotion recognition (i.e. 15–19 participants per condition) [27,28]. Data obtained from 7 participants were affected by technical issues during the recording and therefore were excluded from the analysis.

### Stimuli preparation

In a session prior to the experiment, a set of four pictures (two male and two female) depicting happy, disgusted, angry and neutral expressions was selected from the Karolinska Directed Emotional Faces set [29]. Three sets of stimuli per picture were created with Phantamorph software by morphing each emotional expression with the corresponding neutral expression. This process provided us with one set of 100 morphed photos ranging from 100% neutral to 100% emotional, for each of the three expressions, for each model. Five different strengths of each emotion were selected from each set, comprising 15%, 20%, 30%, 50% and 70%. This provided us with a wide range of stimulus difficulty. The decision of selecting this range of emotional strengths was based on a previous study where a similar procedure was used to create the experimental stimuli for an emotion recognition task [27].

To evaluate the arousing power and the valence attributed to each face, in an initial pilot study six raters—who did not participate in the main study—were asked to rate on a 7-point Likert scale (from 0 “Highly calming” to 7 “Highly arousing”) the arousal level of each picture. Similarly, they were asked to rate the valence of each face (from -3 “Highly negative” to +3 “Highly positive”).

In order to test the significant difference in arousal and valence for the different emotional faces, we run two separate ANOVAs with Emotion (Happiness, Disgust, Anger), Identity (Male1 vs Male2 vs Female1 vs Female 2), and Intensity (15%, 20%, 30%, 50%, 70%) as within-subject factors.

For the arousal rating, a main effect of Intensity was observed [F(4, 20) = 120.47; p < 0.001], showing that the level of perceived arousal progressively increased as the facial emotional intensity increased.

For the valence rating a main effect of Emotion was found [F(2, 10) = 1842.77; p < 0.001], with Happy faces rated as more positive (M = 1.65; SD = 0.08) than Disgusted (M = -1.55; SD = 0.11) [*t*_(5)_ = 47.46; p < 0.001] and Angry faces (M = -1.50; SD = 0.13) [*t*_(5)_ = 41.84; p < 0.001]. A significant Emotion x Intensity interaction was also found [F(8, 40) = 122.30; p < 0.001]. Happy faces were rated progressively more positive as the emotional intensity increased, with the only exception of the first two levels (15%, M = 0.29, SD = 0.37; 20% M = 0.87, SD = 0.26) and the last two levels (50% M = 2.62, SD = 0.31; 70% M = 2.75, SD = 0.22) that did not significantly differ from each other (p > 0.046, Bonferroni corrected).

Disgusted faces were rated progressively more negative as the emotional intensity increased, with the only exception of the 15% (M = -0.21, SD = 0.37) and 20% (M = -0.75, SD = 0.32) levels, the 30% (M = -1.54, SD = 0.33) and 50% (M = -2.33, SD = 0.44) levels and the last two levels (50% M = -2.33, SD = 0.44; 70% M = -2.95, SD = 0.10) that did not significantly differ from each other (p > 0.026, Bonferroni corrected).

Angry faces were rated progressively more negative as the emotional intensity increased, with the only exception of the 30% (M = -1.75, SD = 0.42) and 50% (M = -2.33, SD = 0.30) levels and the last two levels (50% M = -2.33, SD = 0.30; 70% M = -2.75, SD = 0.32) that did not significantly differ from each other (p > 0.042, Bonferroni corrected).

As far as the two food substances were concerned, in an initial pilot study twelve raters—who did not participate in the main study—rated their subjective emotional feeling elicited by the two tastes. Six participants were asked to eat ~5g of dark chocolate and the other six were asked to eat ~5g of fish sauce. After food intake, each participant was asked to rate on two separate Likert scales—from 0, “Not at all”, to 7, “Very strongly”–the extent to which their current emotional state was happy or disgusted, respectively. The pilot study confirmed that the dark chocolate was able to induce a happy emotional state, whereas fish sauce evoked a disgusted reaction. Importantly, the two food substances were equally effective in inducing the expected emotional states.

### Design

The design of the experiment was a 2x3x2 mixed design. The between-subjects factor was the food substance that participants were asked to consume and that was either ~5g of dark chocolate or a teaspoon of fish sauce (~5g). The two within-subjects factors were the facial emotional expression that was displayed in each trial (i.e., happiness, anger or disgust) and the timing of the emotion recognition task (i.e. before or after food intake).

### Task

In an initial session, participants were asked to complete several questionnaires and to perform different ratings, in order to control for the effects of any potential confounding variables.

In order to assess individual disgust sensitivity threshold, participants were asked to complete the Disgust Scale, Version 1 [30]. Higher scores indicate that a person is more disgust sensitive than average.

Moreover, given that the presence of an eating disorder might affect the individual experience of food-induced disgust or pleasantness, we also measured the participants’ risk of presenting an eating disorder, by administering the Eating Attitude Test (EAT-26) [31]. According to the Scoring and Interpretation procedure, this questionnaire alone does not yield a specific diagnosis of an eating disorder, but can be considered a useful screening tool to assess “eating disorder risk”. In particular, individuals who score 20 or more could meet the criteria for an eating disorder, although only an interview with a qualified professional could provide confirmation of that.

Additionally, in order to control for the participants’ appetite at the moment of the experiment, participants were also asked to report their appetite level on a 7-point Likert scale from 0 “Not at all” to 7 “Very much”.

As demonstrated by [32], subjective disgust sensitivity can be predicted by the Body Mass Index (BMI); therefore, at the beginning of the experiment we asked participants to report their weight and height in order to calculated their BMI.

In order to assess the perceived closeness to a person showing a specific emotional expression, participants were asked to watch a sequence of three pictures of a face depicting either a happy, disgusted or angry expression (selected from Karolinska Directed Emotional Faces set and different from those used in the main experiment) and then to indicate, for each of them, how close they felt towards that person by means of a 7-point scale version of the Inclusion of Other in the Self (IOS) scale [33]. The identity and the presentation order of the three emotional faces were randomized across participants. At the end of the experiment participants were asked to perform again the same rating.

Finally, in order to assess the effect of the food substances on the participants’ mood, we asked them to perform an emotion intensity judgment on two Visual Analogue Scales (VAS, from 0cm “Not at all” to 16cm “Very much”) where they had to report their perceived levels of Happiness and Disgust, respectively. Responses were made by drawing a cross on the scale printed on a white sheet. This assessment was repeated also immediately after food ingestion and at the end of the experiment.

The order of the questionnaires and ratings was randomized across participants. This initial session lasted approximately 15 minutes.

Participants were then asked to perform an emotion recognition task before and after consuming either a piece of chocolate or a small amount of fish sauce. The emotion recognition task consisted of 60 trials, each of which displayed either a male or a female model with one of three emotional expressions (anger, happiness or disgust), at one of the five intensity levels (15%, 20%, 30%, 50% or 70%). Each image was presented twice. The order of the trials was randomized. The image remained on the screen for 500 msec, followed by an inter-stimulus interval of the variable duration of 2000-2500-3000 msec. Participants were asked to choose which of the three emotions (anger, disgust or happiness) was displayed, making a 3-alternative forced choice by pressing the corresponding labelled key on the keyboard. The assignment of each face to the different experimental sessions and the assignment of each key to the different emotions were counterbalanced across participants. Each emotion recognition task lasted approximately 5 minutes.

Before the task, in order to familiarize with the position of the response keys, participants completed twelve practice trials where the labels “happiness”, “anger” or “disgust” were presented in the centre of the screen for 1 sec, in random order, and participants were asked to press the corresponding key.

### Procedure

Participants were asked to sit at a distance of ~ 60cm in front of a computer screen and read the information sheet along with an information paper including all the ingredients present in the two food substances. Only if participants reported no allergy or intolerance to any of the ingredients, they were allowed to carry on with the study. Each participant was randomly assigned to either one or the other experimental condition (i.e. dark chocolate or fish sauce group).

The experiment was conducted on a Dell PCs, with high-resolution 17” colour monitor, running E-Prime Professional 2. The main experimental session was divided in three parts: (1) emotion recognition task-Pre; (2) Food intake; (3) emotion recognition task-Post.

Before the main experimental session, participants filled out all the required questionnaires and completed the different ratings scales. Then they performed the emotion recognition task. Those in the chocolate group were then asked to ingest a piece of dark chocolate (~5g) whilst participants in the fish sauce group were asked to ingest a spoonful of fish sauce (~5g). Finally, participants performed for the second time the emotion recognition task where different models were used. The entire experiment took approximately 45 minutes (see Fig 1 for a visual representation of the Procedure).

### Data analysis

First, trials in which participants failed to respond (4%) were discarded from the analysis. Then, the mean Reaction Times (RTs) of the correct responses was calculated for each condition; responses longer than 2.5 standard deviations from the individual mean were treated as outliers and not considered (3%). Percentage of correct responses and RTs in recognizing the three different emotions before and after food ingestion were then entered into two separate three-way Mixed-factor ANOVAs with Time (Pre and Post food intake) and Emotion (Happiness, Disgust and Anger) as the within-subjects factors and Food (Chocolate and Fish sauce) as the between-subjects factor. In case of significant three-way interactions, planned comparisons were conducted.

In order to measure the effectiveness of our experimental manipulation in inducing a happy mood after eating chocolate and a disgusted mood after eating fish sauce, the scores obtained on the VAS were entered into a three-way Mixed-factor ANOVA with Time (Pre experiment, Post food intake and Post experiment) and Emotion (Happiness and Disgust) as the within-subjects factors and Food (Chocolate and Fish sauce) as the between-subjects factor.

In order to test whether (a) individual disgust sensitivity threshold, (b) individual eating disorders risk or (c) BMI could play a modulatory role in the observed effects, for each group of participants (chocolate, fish sauce) percentage of correct responses and RTs were entered into six separate ANCOVAs with Time (Pre and Post food intake) and Emotion (Happiness, Disgust and Anger) as the within-subjects factors and one of the three above mentioned scores—i.e. disgust sensitivity, eating disorder risk, BMI—as covariate.

In order to measure any changes in the perceived closeness to the emotional face depicted in the picture, the ratings obtained on the IOS scale were entered into a three-way Mixed-factor ANOVA with Time (Pre and Post experiment) and Emotion (Happiness, Disgust and Anger) as the within-subjects factors and Food (Chocolate and Fish sauce) as the between-subjects factor.

Finally, in order to control for differences in the participants’ level of appetite at the time of the experiment, an independent-sample *t-test* was run on the appetite scores between the chocolate and the fish sauce group.

## Results

In order to study the effect of food type on emotion recognition ability, we compared participants’ accuracy and RTs in identifying the three different facial emotional expressions (Happiness, Disgust, Anger), before and after food intake. Percentage of correct responses and RTs, were entered in two separate three-way Mixed-factor ANOVAs with Time (Pre and Post food intake) and Emotion (Happiness, Disgust and Anger) as the within-subjects factors and Food (Chocolate and Fish sauce) as the between-subjects factor.

### Percentage of correct responses

**Fig 2:**
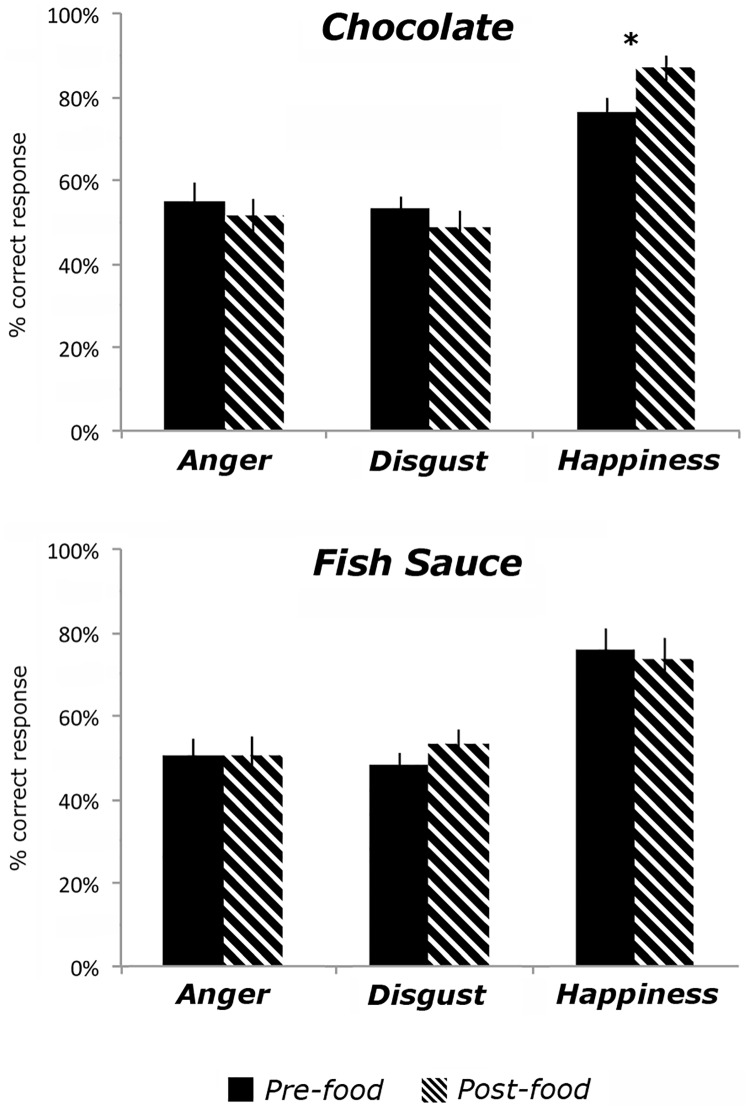
Results for the Emotion Recognition Task. Graphs showing performance on the emotion recognition task, before and after either dark chocolate (top panel) or fish sauce (bottom panel) intake. Accuracy in recognizing angry, disgusted or happy facial expressions were measured (percentage of correct responses, y axis). Error bars reflect standard error of the mean, and asterisk indicates p < 0.05, two-tailed.

For the percentage of correct responses a main significant effect of Emotion was found [F_(2, 82)_ = 51.22; *p* < 0.001; η_p_^2^ = 0.55]. In particular, post-hoc paired sample *t-tests* showed higher accuracy in recognizing happy (M = 78%; sem = 3%) as compared to disgusted (M = 51%; sem = 2%) [*t*_(42)_ = 10.15; p < 0.001; *d* = 1.59] and angry expressions (M = 52%; sem = 3%) [*t*_(42)_ = 8.13; p < 0.001; *d* = 1.23], whereas no difference was observed between disgusted and angry expressions [*t*_(42)_ = 0.33; p = 0.74]. A significant three-way interaction was also found [F_(2, 82)_ = 4.61; *p* = 0.013; η_p_^2^ = 0.10]. Given that our main prediction was that chocolate and fish sauce intake would have specific effects in recognizing happy and disgusted facial expressions respectively, but no significant effect in the ability to recognize angry expressions, we ran planned comparisons between accuracy scores obtained in recognizing each emotion, before and after consuming either food substance. After chocolate intake (M = 87%, sem = 3%), participants were more accurate in recognizing happy expressions as compared to before (M = 76%, sem = 3%), [*t*_(20)_ = 2.80; p = 0.011; *d* = 0.58] whereas no changes were observed for disgusted [*t*_(20)_ = 1.51; p = 0.15] and angry expressions [*t*_(20)_ = 0.92; p = 0.37]. However, after fish sauce intake (M = 53%, sem = 3%), participants did not show any changes in recognizing disgusted (M = 48%, sem = 3%), [*t*_(21)_ = 1.93; p = 0.067; *d* = 0.38], happy [*t*_(21)_ = 0.56; p = 0.58] and angry expressions [*t*_(20)_ = 0.06; p = 0.95], as compared to before (see Fig 2).

### Reaction times

As far as the speed in facial expression recognition was concerned, the main effect of Emotion [F_(2, 82)_ = 41.88; *p* < 0.001; η_p_^2^ = 0.51] was significant. In particular, post-hoc paired sample *t-tests* showed faster recognition of happy (M = 779.18 ms; sem = 29.41) as compared to disgusted (864.43 ms; sem = 38.38) [*t*_(41)_ = 4.52; p < 0.001; *d* = 0.70] and angry expressions (M = 952.83 ms; sem = 43.28) [*t*_(41)_ = 7.71; p < 0.001; *d* = 1.19]. Finally, recognition of disgusted expressions was faster than recognition of angry expressions [*t*_(41)_ = 5.52; p < 0.001; *d* = 0.85]. Interestingly, a significant Time x Emotion x Food interaction [F_(2, 82)_ = 5.55; *p* = 0.006; η_p_^2^ = 0.12] confirmed the expected double dissociation in the effect of food substances on emotion recognition. In order to test our main prediction that chocolate and fish sauce intake would have specific effects in recognizing happy and disgusted facial expressions respectively—but no significant effect in the ability to recognize angry expressions—we ran planned comparisons between RTs obtained in recognizing each emotion, before and after consuming either food substance.

**Fig 3:**
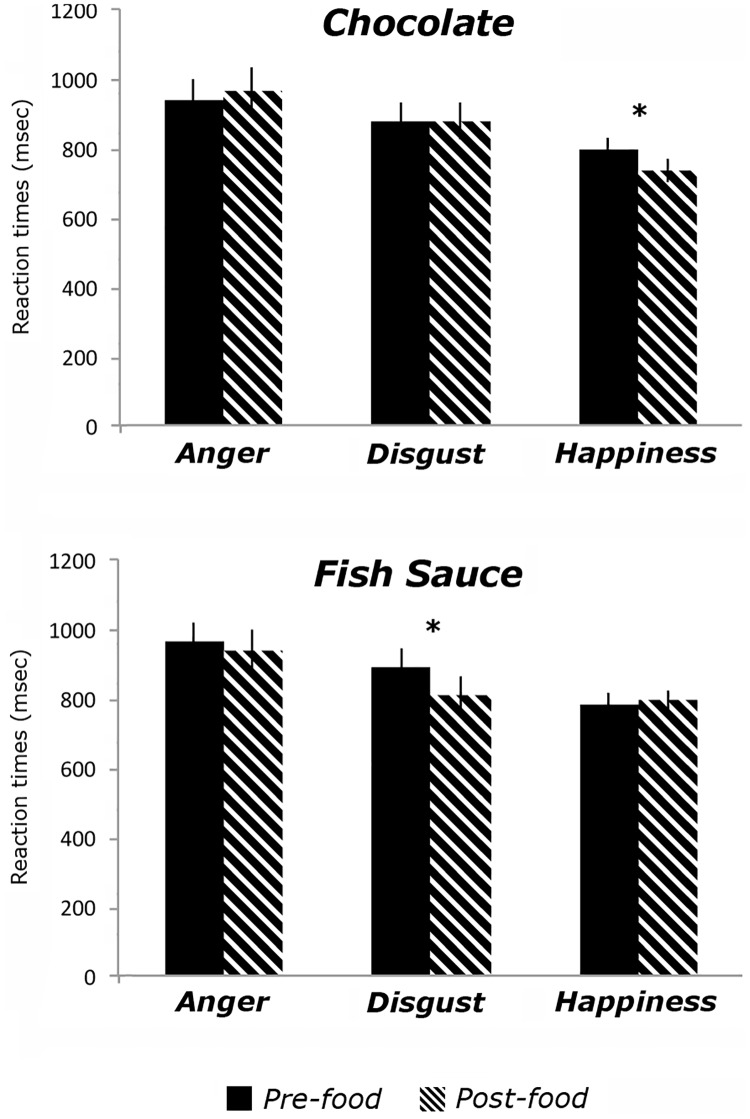
Results for the Emotion Recognition Task. Graphs showing performance on the emotion recognition task, before and after either dark chocolate (top panel) or fish sauce (bottom panel) intake. Mean Reaction Times (msec, y axis) in recognizing angry, disgusted or happy facial expressions were measured. Error bars reflect standard error of the mean, and asterisk indicates p < 0.05, two-tailed.

After chocolate intake (M = 734.47 ms, sem = 33.06), participants were faster in recognizing happy expressions as compared to before (M = 794.42 ms, sem = 36.38) [*t*_(20)_ = 3.31; p = 0.003; *d* = 0.72], whereas no changes were observed for disgusted [*t*_(20)_ = 0.019; p = 0.98] and angry expressions [*t*_(17)_ = 0.79; p = 0.44]. Further, and confirming the double dissociation, participants were faster in recognizing disgusted expressions after consuming a small amount of fish sauce (M = 808.81 ms, sem = 57.48) as compared to before (M = 892.25 ms, sem = 60.98) [*t*_(21)_ = 3.15; p = 0.005; *d* = 0.67], whereas no changes were observed for happy [*t*_(21)_ = 0.52; p = 0.60] and angry expressions [*t*_(21)_ = 0.76; p = 0.45] (see Fig 3).

### Effectiveness of the experimental manipulation

In order to measure the effect of the two food substances on the perceived individual levels of happiness and disgust, we compared participants’ ratings about their subjective emotional feeling at the beginning of the experiment, immediately after eating either a piece of dark chocolate or a spoonful of fish sauce and at the end of the study. The levels on the VAS corresponding to the participants’ subjective feeling were entered into a three-way Mixed-factor ANOVA with Time (Pre experiment, Post food intake, Post experiment) and Emotion (Happiness and Disgust) as the within-subjects factors and Food (Chocolate and Fish sauce) as the between-subjects factor. A main effect of Time was found [F_(2, 82)_ = 19.72; *p* < 0.001; η_p_^2^ = 0.33]. In particular, participants perceived a higher emotional level after food intake (M = 7.75cm, sem = 0.28) and at the end of the experiment (M = 7.50cm, sem = 0.28) than at the beginning of the study (M = 6.16cm, sem = 0,28), [*t*_(42)_ = 4.80; p < 0.001; *d* = 0.72] and [*t*_(42)_ = 4.56; p < 0.001; *d* = 0.69] respectively, whereas no difference was observed between the perceive emotional state after food intake and at the end of the experiment [*t*_(42)_ = 0.94; p = 0.35]. A main effect of Emotion [F_(1, 41)_ = 62.27; *p* < 0.001; η_p_^2^ = 0.60] showed an overall higher subjective feeling of happiness (M = 9.45cm, sem = 0.44) than disgust (M = 4.83cm, sem = 0.54). Interestingly, a significant Time x Emotion x Food interaction was found [F_(2, 82)_ = 43.94; *p* < 0.001; η_p_^2^ = 0.52]. Two separate ANOVAs where run for each Food group with Time (Pre experiment, Post food intake, Post experiment) and Emotion (Happiness and Disgust) as the within-subjects factors. For the group of participants who ate chocolate, a main effect of Emotion was found [F_(1, 20)_ = 116.89; *p* < 0.001; η_p_^2^ = 0.85], showing higher level of happiness (M = 11.20cm, sem = 0.37) than disgust (M = 2.38, sem = 0.63). Also a significant Time x Emotion interaction was found [F_(2, 40)_ = 4.01; *p* = 0.026; η_p_^2^ = 0.17]. Post-hoc paired sample *t-tests* showed that participants reported higher level of happiness immediately after food intake (M = 11.44cm, sem = 0.46) and at the end of the experiment (M = 12.27cm, sem = 0.45) as compared to the beginning of the experiment (M = 10.12cm, sem = 0.63), [*t*_(20)_ = 2.16; p = 0.043; *d* = 0.47] and [*t*_(20)_ = 3.17; p = 0.005; *d* = 0.69] respectively.

For the group of participants who ate fish sauce a main effect of Time was found [F_(2, 42)_ = 27,67; *p* < 0.001; η_p_^2^ = 0.57], showing higher emotional feeling after food intake (M = 8.69cm, sem = 0.37) and at the end of the experiment (M = 7.76cm, sem = 0.44) as compared to before (M = 5.86cm, sem = 0.34), [*t*_(21)_ = 7.38; p < 0.001; *d* = 1.57] and [*t*_(21)_ = 4.41; p < 0.001; *d* = 0.94] respectively. Moreover participants reported higher emotional level immediately after food intake than at the end of the experiment [*t*_(21)_ = 2.70; p = 0.013; *d* = 0.57]. Importantly, also a significant Time x Emotion interaction was found [F_(2, 42)_ = 47.93; *p* < 0.001; η_p_^2^ = 0.69]. Post-hoc paired sample *t-tests* showed that participants reported higher level of disgust immediately after food intake (M = 12.21cm, sem = 0.89) and at the end of the experiment (M = 7.8cm, sem = 0.71) as compared to the beginning of the experiment (M = 1.48, sem = 0.51), [*t*_(21)_ = 11.22; p < 0.001; *d* = 2.39] and [*t*_(21)_ = 8.46; p < 0.001; *d* = 1.80] respectively. Moreover participants reported higher level of disgust immediately after food intake as compared to the end of the experiment [*t*_(21)_ = 4.09; p = 0.001; *d* = 0.87]. Interestingly, in this group of participants also a change in the level of perceived happiness was observed. In particular, participants reported to be happier at the beginning of the experiment (M = 10.23cm, sem = 0.66) than immediately after eating the fish sauce (M = 5.17cm, sem = 0.78) and at the end of the experiment (M = 7.72cm, sem = 0.83), [*t*_(21)_ = 6.21; p < 0.001; *d* = 1.32] and [*t*_(21)_ = 3.34; p = 0.003; *d* = 0.71] respectively.

### Disgust sensitivity

In order to control for any modulatory effects of individual disgust sensitivity threshold on the observed effects, for each group of participants percentage of correct responses and RTs were entered into two separate ANCOVAs the with Time (Pre and Post food intake) and Emotion (Happiness, Disgust and Anger) as the within-subjects factors and Disgust Sensitivity scores, as covariate.

For both groups of participant, both the percentage of correct responses and RTs showed neither significant main effects nor interactions (all p > 0.09).

### Eating disorder risk

In order to control for any modulatory effects of individual eating disorder risks on the observed effects, for each group of participants percentage of correct responses and RTs were entered into two separate ANCOVAs the with Time (Pre and Post food intake) and Emotion (Happiness, Disgust and Anger) as the within-subjects factors and EAT-26 scores, as covariate.

For both groups of participant, both the percentage of correct responses and RTs showed neither significant main effects nor interactions (all p > 0.06).

### BMI

In order to control for any modulatory effects of individual BMI on the observed effects, for each group of participants percentage of correct responses and RTs were entered into two separate ANCOVAs the with Time (Pre and Post food intake) and Emotion (Happiness, Disgust and Anger) as the within-subjects factors and BMI, as covariate.

For both groups of participant, both the percentage of correct responses and RTs showed neither significant main effects nor interactions (all p > 0.16).

### IOS scale

In order to measure any changes in the perceived closeness to an emotional face as a function of our experimental manipulation, the ratings obtained on the IOS scale were entered into a three-way Mixed-factor ANOVA with Time (Pre and Post experiment) and Emotion (Happiness, Disgust and Anger) as the within-subjects factors and Food (Chocolate and Fish sauce) as the between-subjects factor. A main effect of Emotion was found [F_(2, 82)_ = 104.12; *p* < 0.001; η_p_^2^ = 0.72]. Participants felt in general closer to the happy faces (M = 4.5, sem = 0.22) than to the disgusted (M = 1.96, sem = 0.16) and angry ones (M = 1.47, sem = 0.09), [*t*_(42)_ = 8.18; p < 0.001; *d* = 1.24] and [*t*_(42)_ = 12.12; p < 0.001; *d* = 1.84] respectively. Moreover, participants felt closer to the disgusted than the angry faces [*t*_(42)_ = 3.54; p = 0.001; *d* = 0.53]. Importantly, a significant interaction was found between Time, Emotion and Food [F_(2, 82)_ = 10.50; *p* < 0.001; η_p_^2^ = 0.20]. For each group of participants a Repeated Measure ANOVA with Time (Pre and Post experiment) and Emotion (Happiness, Disgust and Anger) as the within-subjects factors was run.

For the participants who ate chocolate, a main effect of Emotion was found [F_(2, 40)_ = 100.85; *p* < 0.001; η_p_^2^ = 0.83], showing an overall feeling of being closer to the happy faces (M = 4.88, sem = 0.29) than to the disgusted (M = 1.47, sem = 0.15) and the angry ones (M = 1.19, sem = 0.07), [*t*_(20)_ = 8.94; p < 0.001; *d* = 1.95] and [*t*_(20)_ = 12.15; p < 0.001; *d* = 2.65] respectively.

Similarly, for the participants who ate fish sauce, a main effect of Emotion was found [F_(2, 42)_ = 25.52; *p* < 0.001; η_p_^2^ = 0.55], showing an overall feeling of being closer to the happy faces (M = 4.15, sem = 0.34) than to the disgusted (M = 2.43, sem = 0.24) and the angry ones (M = 1.75, sem = 0.16), [*t*_(21)_ = 4.05; p = 0.001; *d* = 0.86] and [*t*_(21)_ = 6.87; p < 0.001; *d* = 1.46] respectively. Moreover, participants felt closer to the disgusted than the angry faces [*t*_(21)_ = 2.83; p = 0.010; *d* = 0.60]. Importantly, for this group of participants also a significant Emotion x Time interaction was found [F_(2, 42)_ = 11.02; *p* < 0.001; η_p_^2^ = 0.34]. In particular, participants felt closer to disgusted faces after (M = 3.13, sem = 0.34) than before (M = 1.72, sem = 0.25) the experiment [*t*_(21)_ = 3.74; p = 0.001; *d* = 0.79]. Conversely, after the experiment (M = 3.55, sem = 0.39) participants felt less close to happy faces than before (M = 4.77, sem = 0.38) [*t*_(21)_ = 3.15; p = 0.005; *d* = 0.67]. No change in perceived closeness to angry face was found [*t*_(21)_ = 1.47; p = 0.16].

### Appetite

An independent-sample *t-test* was run on the Appetite scores in order to control for the appetite level across the two groups of participants. The results showed that, at the time of the experiment, the two groups of participants did not significantly differ in terms of their appetite level [*t*_(41)_ = 1.31; p = 0.19].

## Discussion

With the present study, we tested whether the simulation mechanism automatically activated when observing someone else’s emotional expression requires the observer *to share* the inferred emotion with the other person in order to better understand the other’s emotional state. In particular, we investigated whether recognition of specific emotional expressions improves when the observer’s own emotional state resonates with them.

Our results demonstrated that this simulation mechanism is actually facilitated if we share the observed emotional state. In particular, after eating chocolate—which has been repeatedly found to elevate mood [24,25]–participants showed an improved ability to recognize happy facial expressions, whereas those who consumed a highly disgusting food substance—i.e. fish sauce—were significantly faster at detecting disgusted expressions. Therefore, increasing interpersonal emotional resonance—i.e. experiencing the same emotional state as someone else—seems to foster the remapping of the observed experience onto one’s own sensorimotor system, boosting the automatic simulation process and eventually facilitating emotional understanding.

The crucial role of interpersonal resonance in better understanding others’ experiences and feelings has been widely demonstrated in social psychology studies, which have shown that synchronic shared experiences (e.g. rituals, marching) enhance social cohesion by relaxing the psychological boundaries between individuals [34]. More recently, it has been shown that shared multisensory experiences between people (e.g. synchronous, but not asynchronous, tactile stimulation on one’s face while viewing another face being touched) can increase the sense of merging with the other [35,36]. Importantly, it has been recently suggested that this increased self-other merging facilitates inference of the other’s physical and mental states [36–38]. Therefore, in the present study, we tested whether the ability to infer others’ states is associated with changes in perceived self-other distance. In particular, we investigated whether reducing the emotional distance between people—i.e. by enhancing their emotional resonance—would eventually facilitate the simulation mechanism, improving understanding of the other person’s feelings. Our results showed that the improved ability to recognize disgust induced by fish sauce ingestion was associated with higher closeness ratings for disgusted faces. This demonstrates that sharing the same disgusted emotional state with someone else reduces the perceived distance from that person. However, this effect was not observed for happy faces after eating chocolate. A possible explanation for the lack of an increased sense of closeness to a happy face after eating chocolate could be that participants’ ratings of their initial level of happiness were already quite high at the beginning of the experiment. Therefore any increases in positive mood following our experimental manipulation might not have been strong enough to induce a noticeable effect.

The majority of the previous studies on emotion recognition used paradigms based exclusively on vision. However, the process of recognizing emotional facial expressions relies not only on visual exteroceptive information, but also on non-visual interoceptive signals arising from the observer’s own internal states [39,40]. Interestingly, food-mood interactions have been widely investigated and the effects of different food substances on emotional states have been consistently demonstrated [23], providing clear evidence that taste and odours are strong and reliable elicitors of emotional reactions and the related facial expressions [22,41,42]. For example, Macht and Dettmer demonstrated that participants’ positive mood increased after eating chocolate or an apple, with a stronger and longer-lasting effect after chocolate intake [24]. Our results confirmed the effectiveness of specific food substances in inducing particular emotional reactions, as our participants’ mood ratings after eating chocolate or fish sauce showed a significant increase in happiness or disgust, respectively.

In line with the assumption that taste can effectively induce emotional reactions, the present study tested—and confirmed—the hypothesis that recognizing an emotion from an observed facial expression does not solely depend on visual processing, but also relies strongly on the observer’s internal state.

Recent studies on 3-month old infants showed that after the babies inhaled a pleasant odour—such as strawberry—they spent more time looking towards a happy face than a disgusted one [22]. According to Godard and colleagues, the odour might have elicited a pleasant feeling, eventually activating facial muscles normally recruited when displaying that positive emotional state. The activation of emotion-specific facial muscles might have consequently boosted the automatic imitational system [43], whereby infants tend to always imitate the facial expressions presented in front of them. Therefore, it was suggested that the odour would have oriented the infants’ gaze towards the expression that was compatible with their “pre-mobilized” facial muscles [22].

### Potential role of confounding variables

Several variables could have interacted with the mechanism under investigation. The first important variable we controlled for was the subjective disgust sensitivity threshold. A lower threshold might have been responsible for the observed enhancement of disgust recognition in the group of participants who ate fish sauce. Therefore, at the beginning of the experiment, participants were asked to fill out the “Disgust Scale” [30] in order to assess their disgust sensitivity. The results showed that disgust sensitivity did not vary between the two groups, and, thus, did not interact with the observed effects.

Importantly, it has been shown that disgust sensitivity can be strongly affected by BMI. In particular, people with higher BMI show a decreased level of core disgust that could be responsible for their increased tendency to overeat [32]. In the present study, we also controlled for this variable, and we did not find any correlation between participants’ BMI and their increased emotion recognition ability following food ingestion.

In addition, the presence of eating disorders has been found to highly correlate with disgust sensitivity. Aharoni and Hertz [44] demonstrated that individuals suffering from Anorexia Nervosa scored consistently higher on the Disgust Scale [30]. Therefore, we assessed participants’ risk of presenting an eating disorder by means of the EAT-26 [31]. We did not find any interaction between this index and our effect of interest.

## Conclusion

To conclude, our findings suggest that participants’ automatic simulation of the observed facial expression might have been preferentially facilitated by a congruent emotional state, induced by food consumption. Thus, their perception of their own internal feelings may have promoted and improved their recognition of the corresponding facial expression observed in the models. Importantly, for the first time, we have provided direct evidence of the critical role played by this *emotion sharing mechanism* in understanding others’ emotional states.

Given that the present study is psychophysical, future electromyographic or neuroimaging studies will be required to provide clear confirmation of the suggested simulation mechanism.

## Supporting Information

S1 Data (SAV)
